# Bilateral Adrenal Hemorrhage and Acute Adrenal Insufficiency Secondary to Heparin-Induced Thrombocytopenia After Coronary Artery Bypass Graft Surgery

**DOI:** 10.1016/j.aed.2025.08.009

**Published:** 2025-08-22

**Authors:** Heng Yeh, Suprita Shrestha, Katrina Ann H. Corpuz, Suman Paudel

**Affiliations:** Bassett Medical Center, Department of Medicine, Cooperstown, New York

**Keywords:** adrenal insufficiency, bilateral adrenal hemorrhage, heparin-induced thrombocytopenia, coronary artery bypass graft surgery

## Abstract

**Background/Objective:**

Adrenal insufficiency (AI) secondary to bilateral adrenal hemorrhage (BAH) from heparin-induced thrombocytopenia (HIT) is rare; only 3 instances of such cases were reported after coronary artery bypass graft surgery (CABG). We report a case of AI due to BAH from HIT after an elective CABG.

**Case Description:**

A 56-year-old male with a history of diabetes and multivessel coronary artery disease underwent CABG a week ago presented with abdominal pain. Labs showed leukocytosis, hyperglycemia, elevated anion gap, and positive serum ketones. Intravenous insulin with empirical antibiotics was started. Platelet decline was noticed from 154 000/uL to 39 000/uL on the following days, along with fever, hypotension, altered mentation, hyponatremia, and abdominal pain. A computed tomography scan of the abdomen showed BAH. Primary AI was confirmed with a cosyntropin stimulation test, and HIT was diagnosed with Heparin-PF4 IgG assay and serotonin release assay. His symptoms improved after hydrocortisone treatment.

**Discussion:**

AI secondary to BAH from HIT is a rare complication, with most cases reported after orthopedic surgeries. Symptoms of AI are vague and similar to sepsis. If hyponatremia, and gastrointestinal symptoms are noticed, adrenal insufficiency should be suspected, and early stress dose steroid treatment should be considered. Steroid treatment in such cases has shown to improve survival.

**Conclusion:**

The symptoms of adrenal insufficiency are nonspecific and could be similar to sepsis. Electrolyte abnormalities and gastrointestinal symptoms could be hints for adrenal insufficiency, especially if risks for adrenal hemorrhage are present. Early stress dose treatment in such cases has shown a survival benefit.

**Highlights**

• Bilateral adrenal hemorrhage secondary to heparin-induced thrombocytopenia is a rare but lethal complication. Most cases were reported after orthopedic surgeries, but could also occur in cardiac surgery
• When bilateral adrenal hemorrhage is suspected, adrenal function tests are mandatory due to the risk of primary adrenal insufficiency
• The most common symptoms of adrenal insufficiency are vague and similar to sepsis, including fever, altered mentation, and labile blood pressure. Early diagnosis requires high index of suspicion. Abdominal pain and hyponatremia are common reported symptoms in adrenal insufficiency
• Treatment of heparin-induced thrombocytopenia-associated adrenal insufficiency is similar to primary adrenal insufficiency. Early corticosteroid treatment has been shown to improve survival in adrenal hemorrhage patients, especially after surgery

**Clinical Relevance**

We believe this case report provides valuable insights for endocrinologists and treating physicians in the early recognition and treatment of adrenal insufficiency secondary to bilateral adrenal hemorrhage, especially in high-risk patients present with septic symptoms, abdominal pain, and electrolyte abnormalities.

## Introduction

Heparin-induced thrombocytopenia (HIT) is an immune complication caused by antibodies against immune complexes formed by platelet factor 4 (PF4) and heparin, which subsequently bind to platelet and monocyte Fc receptors, promoting procoagulant microparticle release and thrombin generation.^1^ In patients undergoing cardiac surgery, the incidence of HIT antibody seroconversion approaches close to 50%, potentially secondary to the high intraoperative unfractionated heparin (UFH) doses on the cardiopulmonary bypass.^1^^,^^2^ However, the incidence of clinical HIT in cardiac surgery patients only ranges from 1% to 5%.^3^

Bilateral adrenal hemorrhage (BAH) induced by HIT is a rare but significant complication, primarily observed in patients who underwent orthopedic procedures and were associated with UFH.^4^ The adrenal glands have up to 60 arterioles but only a single central vein for venous drainage, which is susceptible to obstruction in the prothrombotic status of HIT. The subsequent increased capillary pressure due to venous obstruction leads to hemorrhage within the glands.^4^^,^^5^ The mortality rate of BAH and adrenal insufficiency (AI) from HIT could be up to 27.8% and even 100% in undiagnosed patients.^4^ The most common manifestations were fever, abdominal pain, hypotension, and hyponatremia, followed by altered mental status, vomiting, and dizziness.^4^ When BAH is suspected, testing for adrenal function is mandatory due to the risk of primary adrenal insufficiency.^5^

Adrenal insufficiency secondary to HIT associated with cardiac bypass graft surgery (CABG) is very rare. To our knowledge, only 3 other cases have been reported so far.^6^, ^7^, ^8^ We report a case of adrenal insufficiency and bilateral adrenal hemorrhage secondary to HIT after a CABG surgery.

## Case Description

A 56-year-old male with type 2 diabetes mellitus, hypertension, and a history of coronary artery disease with stent placement in the right coronary artery, presented to the hospital with generalized abdominal pain, nausea, vomiting, and diarrhea.

He underwent an elective CABG surgery for a triple-vessel disease found by a nonemergent cardiac catheterization 7 days ago without significant complications. He received UFH during the CABG surgery and enoxaparin for deep vein thrombosis prophylaxis post-operatively. He was discharged 1 day before the current presentation.

He was afebrile with elevated blood pressure 157/85 mmHg and exhibited diffuse abdominal tenderness. He was saturating 100% on a nasal cannula flowing at 2 L/min.

**Fig. 1:**
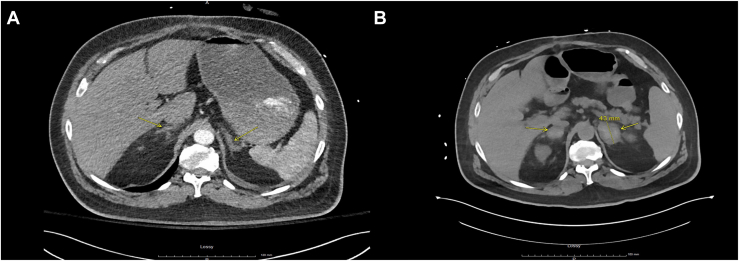
*A*, the initial CT abdomen and pelvis showing normal adrenal glands upon initial presentation. *B*, the CT abdomen and pelvis on the night of thrombocytopenia nadir, showing bilateral heterogenous enlarged adrenal glands representing bilateral adrenal hemorrhage. *CT* = computed tomography.

The initial blood tests are listed in Table 1. These tests revealed severely elevated blood glucose levels, elevated creatinine, elevated beta-hydroxybutyrate, and elevated anion gap with neutrophilic leukocytosis. The computed tomography (CT) abdomen and pelvis (Fig. 1 *A*) showed no acute findings in the abdomen, but left lower lobe consolidation with pleural effusion. He was diagnosed with diabetic ketoacidosis and started with normal saline and intravenous insulin infusion. Empirical cefepime and vancomycin were initiated for the treatment of hospital-acquired pneumonia. Subcutaneous enoxaparin was given daily for deep vein thrombosis prophylaxis.

**Table 1:** Pertinent Blood Test Results on the Day of Admission, the Day of Thrombocytopenia Nadir, and the Day of Discharge

Test name	Initial lab tests on the day of admission	Lab tests on the day of the thrombocytopenia nadir	Lab tests on the day of discharge
Sodium 136-145 mmol/L	135 (L)	130 (L)	132 (L)
Potassium 3.4-4.5 mmol/L	3.7	4.0	4.3
Chloride 98-110 mmol/L	93 (L)	97	102
Glucose random 70-139 mg/dL	371 (H)	86	223 (H)
Co2 21-31 mmol/L	22	23	23
Anion gap 6-14 mmol/L	20 (H)	10	7
BUN 7-25 mg/dL	37 (H)	33 (H)	43 (H)
Creatinine 0.7-1.3 mg/dL	1.5 (H)	1.6 (H)	1.3
Calcium 8.6-10.3 mg/dL	9.5	8.0 (L)	7.9 (L)
Beta-hydroxybutyrate 0.02-0.27 mmol/L	2.60 (H)	-	
Alkaline phosphatase 34-104 U/L	70	-	
Albumin 3.5-5.7 g/dL	4.3	-	
Albumin/globulin ratio 1.1-2.1	1.3	-	
Total protein 6.0-8.3 g/dL	7.7	-	
AST 13-39 U/L	19	-	
ALT 7-72 U/L	16	-	
Total bilirubin 0.3-1.0 mg/dL	0.9	-	
Lipase 11-82 U/L	27	-	
White blood cell count 3.7-10.6 × 10^3^ cells/uL	21.4 (H)	16.2 (H)	13.3 (H)
RBC 3.70-5.90 × 10^6^ cells/uL	3.92	3.56 (L)	3.02 (L)
Hemoglobin 11.5-18.0 g/dL	11.2 (L)	10.3 (L)	8.6 (L)
Hematocrit 35.0-50.0%	33.9%	31.2% (L)	25.8% (L)
MCV 81.0-99.0 fL	86.5	87.6	85.4
Platelet count 140-425 10^3^ cells/uL	219	39 (L)	142
Fibrinogen 162-509 mg/dL	-	449	
INR 0.9-1.2	-	1.4 (H)	
Prothrombin time 10.1-13.2	-	16.0 (H)	
Partial thrombin time 27.1-39.1	-	34.8	
D-dimer Ng/mL FEU	-	104 943 (H)

Twelve hours after insulin treatment, the anion gap normalized, and the abdominal pain improved. The insulin regimen was transitioned to subcutaneous insulin, and the antibiotics were changed to cefpodoxime and azithromycin.

**Fig. 2:**
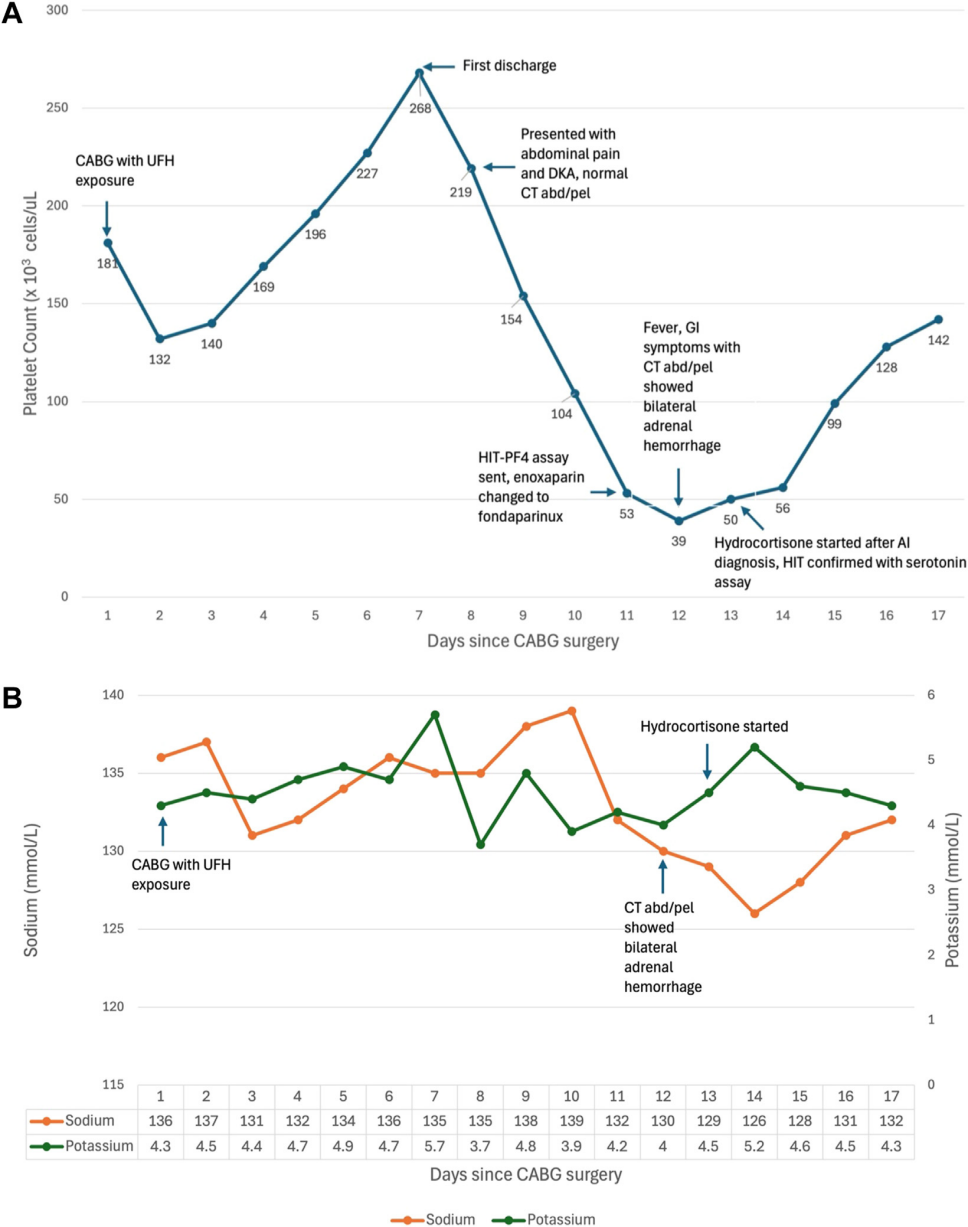
*A*, the trending for platelet counts after CABG surgery with important clinical events. *B*, the trending of sodium and potassium levels after CABG surgery. *CABG* = coronary artery bypass graft; *CT* = computed tomography; *UFH* = unfractionated heparin; *HIT-PF4* = heparin-induced thrombocytopenia-platelet factor 4.

An isolated downtrend in platelet counts was noticed on the following days. The platelet counts dropped from 219,000/uL on day 1 to 53,000/uL on day 4 of hospitalization, with a nadir of 39,000/uL on day 5. No signs of any new bleeding, ecchymosis, or blood clots were noticed. The INR was mildly elevated, and the partial thromboplastin time and fibrinogen were normal. His sodium level also dropped from 135 mmol/L on the day of admission to 130 mmol/L on the day of thrombocytopenia nadir. Other pertinent laboratory data are listed in Table 1, and the trending of the platelet counts and electrolytes are shown in Fig. 2 *A* and *B*.

Given his recent heparin exposure and the isolated thrombocytopenia, type 2 HIT was suspected. The 4T score was 6 points, based on more than 50% of decrease in platelet count with the nadir above 20,000/uL, timing of the thrombocytopenia started on day 9 after initial heparin exposure, and no other identified causes of thrombocytopenia. HIT IgG antibody to platelet factor 4 (PF4) assay was sent, and Enoxaparin was changed to fondaparinux.

On the same night of his nadir, he developed a fever of up to 38.9 °C with altered mental status. He also exhibited dyspnea with an increased respiratory rate, nausea, vomiting, abdominal pain, and watery diarrhea. There was no focal tenderness on the abdomen and no rebound or guarding. His blood pressure readings fluctuated, with mean arterial pressure dropping from an average of 80s to occasional 60s, and required a high-flow nasal cannula.

The antibiotic was changed to piperacillin-tazobactam and vancomycin. Blood culture, stool Clostridium difficile toxin, and gastrointestinal panel were negative. CT head showed no acute findings. CT abdomen and pelvis (Fig. 1 *B*) showed heterogeneous enlargement of bilateral adrenal glands, indicating bilateral adrenal gland hemorrhage. Baseline cortisol, renin, and aldosterone levels are shown in Table 2. The cortisol level failed to increase with 250 mcg cosyntropin stimulation. Stress dose of hydrocortisone was initiated with a 100 mg hydrocortisone bolus followed by 50 mg every 6 hours. Fondaparinux was changed to argatroban to decrease the bleeding risk. The HIT IgG antibody to PF4 assay came positive, followed by a positive serotonin release assay, confirming the diagnosis of type 2 HIT.

**Table 2:** Hormonal Tests for Adrenal Insufficiency Evaluation on the Day of Thrombocytopenia Nadir and After Discharge

Test name	Hormonal tests on the day of thrombocytopenia nadir	Repeat hormonal tests after discharge from the hospital
Cortisol baseline 6.7-22.6 ug/dL	5.3 (L)	6.4 (L) at 3 mo and 5.7 (L) at 1 y
ACTH 7.2-63 pg/mL	-	124 (H)
Cortisol 1 h after 250 ug cosyntropin stimulation <18 ug/dL after stimulation consider insufficiency	6.1 (L)	-
Aldosterone ≤21 ng/dL	<4.0 (L)	<4.0 (L)
Renin activity 2.9-10.8 ng/mL/H while sodium is depleted	17 (H)	1.2 (L)

After starting the hydrocortisone, no further fever episodes were documented, and his blood pressure stabilized with MAP in the average 70s. His sodium level increased from 126 mmol/L to 132 mmol/L. The hydrocortisone dose was gradually tapered from 50 mg every 6 h to 30 mg in the morning and 20 mg in the evening before discharge. His platelet level also improved after discontinuing the enoxaparin.

The patient was evaluated in the clinic 2 weeks after discharge. He reported no salt cravings, no hypotensive episodes, and no orthostatic hypotension, with normal sodium (136 mmol/L) and potassium (4.4 mmol/L). The hydrocortisone dosage was reduced to 20 mg in the morning and 10 mg in the evening. Since there were no clinical signs of mineralocorticoid insufficiency, he was continued on hydrocortisone without adding fludrocortisone. The repeated hormonal tests are listed in Table 2. The low morning cortisol levels and elevated ACTH levels still indicated primary adrenal insufficiency, with normalization of renin activity and aldosterone levels.

## Discussion

Our case shared similar features of previous reported BAH secondary to HIT after a CABG, including fever, hypotension, altered mental status, diffuse abdominal pain, and diarrhea. In the previously reported cases, the onset of thrombocytopenia and symptoms was 5-8 days after initial heparin exposure. All the cases, including ours, had documented adrenal insufficiency with abnormal cosyntropin stimulation test or low morning cortisol.^6^, ^7^, ^8^

Acute adrenal insufficiency (AI) from BAH could be difficult to diagnose in time. Typical symptoms of AI, including fever, hypotension, and abdominal pain, usually lead to the sepsis workup with empirical antibiotic initiation,^4^ which was reported in almost all cases mentioned above.^6^, ^7^, ^8^

Treatment of HIT-associated adrenal insufficiency is similar to primary adrenal insufficiency. For adrenal crisis patients, immediate parenteral hydrocortisone 100 mg followed by 200 mg of hydrocortisone in 24 h with intravenous fluid infusion is recommended.^9^ Switching to oral hydrocortisone and tapering it to physiologic dose is recommended after stabilization, typically 15-25 mg daily in 2-3 divided doses along with fludrocortisone replacement when the dose of hydrocortisone is less than 50 mg daily.^10^ Corticosteroid treatment has been shown to improve survival in adrenal hemorrhage postoperatively and in antiphospholipid antibody syndrome.^11^

Our case was never treated with fludrocortisone due to a lack of clinical and biochemical features of mineralocorticoid deficiency after stabilization with hydrocortisone, including hypotension and normal sodium and potassium levels. Our case also demonstrated a low aldosterone level with normal renin activity at 1-year follow-up. In a case series monitoring long-term adrenal function in 4 patients with adrenal insufficiency from bilateral adrenal hemorrhage, 2 cases were never treated with fludrocortisone and only on hydrocortisone replacement, and the fludrocortisone replacement in the other 2 patients was discontinued with normalization of the electrolytes and recovery in aldosterone function.^12^

Most cases with bilateral adrenal hemorrhage lead to atrophic adrenal glands with irreversible adrenal failure, requiring lifelong glucocorticoid replacement.^10^ However, still a few cases were reported with partial response or normal response to synacthen stimulation or having a normal baseline serum cortisol.^12^^,^^13^ Evaluation of morning cortisol levels every 3 to 6 months in the initial 1 to 2 years after diagnosis is a reasonable approach to reassess the recovery of glucocorticoid function. If the cortisol level is between 5 and 12 ug/dL, a stimulation test is warranted. However, if the level is more than 12 ug/dL, it suggests the recovery of adrenal function and glucocorticoid could be discontinued.^10^

## Conclusion

Adrenal insufficiency and bilateral adrenal hemorrhage secondary to HIT is a rare but lethal complication and requires high clinical suspicion. Electrolyte abnormalities and gastrointestinal symptoms could be hints for adrenal insufficiency in addition to sepsis symptoms, especially if risks for adrenal hemorrhage are present. Early parenteral stress-dose corticosteroids could improve adrenal insufficiency symptoms and survival rates.

## Statement of Patient Consent

Informed consent was obtained from the patient for the publication of this case and associated images.

## Disclosure

The authors have no conflicts of interest to disclose.
